# Direct Oral Anticoagulants as the First Choice of Anticoagulation for Patients with Peripheral Artery Disease to Prevent Adverse Vascular Events: A Systematic Review and Meta-Analysis

**DOI:** 10.3390/jcdd10020065

**Published:** 2023-02-03

**Authors:** Enikő Pomozi, Rita Nagy, Péter Fehérvári, Péter Hegyi, Boldizsár Kiss, Fanni Dembrovszky, Annamária Kosztin, Sándor Nardai, Endre Zima, Zoltán Szeberin

**Affiliations:** 1 Department of Vascular and Endovascular Surgery, Semmelweis University, 1122 Budapest, Hungary; 2 Centre for Translational Medicine, Semmelweis University, 1085 Budapest, Hungary; 3 Heim Pál National Pediatric Institute, 1089 Budapest, Hungary; 4 Institute for Translational Medicine, University of Pécs, 7624 Pécs, Hungary; 5 Department of Biostatistics, University of Veterinary Medicine, 1078 Budapest, Hungary; 6 Division of Pancreatic Diseases, Heart and Vascular Center, Semmelweis University, 1122 Budapest, Hungary; 7 Heart and Vascular Centre, Semmelweis University, 1122 Budapest, Hungary

**Keywords:** peripheral artery disease, atrial fibrillation, oral anticoagulants, bleeding, cardiovascular outcomes, lower-limb complications

## Abstract

The best method of anticoagulation for patients with peripheral artery disease (PAD) is still a topic of interest for physicians. We conducted a meta-analysis to compare the effects of direct oral anticoagulants (DOACs) with those of vitamin-K-antagonist (VKA) anticoagulants in patients with peripheral artery disease. Five databases (Medline (via PubMed), EMBASE, Scopus, Web of Science, and CENTRAL) were searched systematically for studies comparing the effects of the two types of anticoagulants in patients with PAD, with an emphasis on lower-limb outcomes, cardiovascular events, and mortality. In PAD patients with concomitant non-valvular atrial fibrillation (NVAF), the use of DOACs significantly reduced the risk of major adverse limb events (HR = 0.58, 95% CI, 0.39–0.86, *p* < 0.01), stroke/systemic embolism (HR 0.76; 95% CI 0.61–0.95; *p* < 0.01), and all-cause mortality (HR 0.78; 95% CI 0.66–0.92; *p* < 0.01) compared with warfarin, but showed similar risks of MI (HR = 0.81, 95% CI, 0.59–1.11, *p* = 0.2) and cardiovascular mortality (HR = 0.77, 95% CI, 0.58–1.02, *p* = 0.07). Rivaroxaban at higher doses significantly increased the risk of major bleeding (HR = 1.16, 95% CI, 1.07–1.25, *p* < 0.01). We found no significant difference in terms of revascularization (OR = 1.49, 95% CI, 0.79–2.79, *p* = 0.14) in PAD patients in whom a poor distal runoff was the reason for the anticoagulation. DOACs have lower rates of major limb events, stroke, and mortality than VKAs in PAD patients with atrial fibrillation. Rivaroxaban at higher doses increased the risk of major bleeding compared with other DOAC drugs. More high-quality studies are needed to determine the most appropriate anticoagulation regimen for patients with lower-limb atherosclerosis.

## 1. Introduction

Peripheral artery disease (PAD) affects more than 200 million people worldwide and is responsible for a significant proportion of limb losses and even fatal cardiovascular events due to the progression of the underlying atherosclerotic process [1]. Recent standard protocols prescribe antiplatelet therapy after surgical revascularization procedures [2,3,4,5]. The anticoagulation of these patients is still a controversial topic. The most common condition indicating anticoagulation is non-valvular atrial fibrillation (NVAF) [6]. The concomitant presence of atrial fibrillation and symptomatic peripheral artery disease is frequently reported, especially in the older population [7]. These two conditions share several common risk factors, and their concomitant presence increases the risk of cardiovascular mortality and the incidence of major adverse cardiovascular events exponentially [8,9,10,11]. Therefore, it is important to reduce the risk of these complications with the appropriate drug treatment. DOACs have been shown to be superior in efficacy and safety to vitamin-K-antagonist anticoagulants in the prevention of venous thrombotic events [12,13,14,15,16,17,18], but warfarin and coumadin derivatives are still frequently used anticoagulant drugs in NVAF patients. The detrimental effect of long-term vitamin-K-antagonist therapy on the progression of atherosclerosis has been reported, but there are no clear data on whether the DOACs have the same effect [19,20,21,22]. Nowadays, there is still no clear recommendation for anticoagulation in patients with PAD alone and without any other indication for anticoagulation. In our study, we aimed to determine whether there was a difference in the clinical endpoints in patients with PAD taking direct oral anticoagulants compared with patients taking conventional vitamin-K antagonists. We performed a systematic review and meta-analysis to evaluate the efficacy and safety of DOACs compared with VKA therapy in patients with NVAF and PAD, focusing on mortality, major adverse limb events, major adverse cardiovascular events, and major bleeding.

## 2. Materials and Methods

This systematic review and meta-analysis was conducted according to the Preferred Reporting Items for Systematic Reviews and Network Meta-Analysis (PRISMA-NMA) statement and following a protocol preregistered at the international prospective register of systematic reviews (PROSPERO) with the registration number CRD42021288677.

### 2.1. Search Strategy and Data Sources

We conducted a comprehensive literature search on databases including Medline (via PubMed), EMBASE, Scopus, Web of Science, and CENTRAL with the following search key: (“peripheral artery disease” OR “peripheral arterial disease” OR “PAD” OR “lower extremity arterial disease” OR “lower extremity artery disease” OR “lower-extremity arterial disease” OR “DLEAD” OR “PAOD” OR “POAD” OR “PVOD” OR “PVD”) AND (“novel oral” OR “NOAC” OR “direct oral” OR “DOAC” OR “dabigatran” OR “apixaban” OR “rivaroxaban” OR “edoxaban” OR “non-vitamin K antagonist” OR “non vitamin K antagonist” OR “direct thrombin” OR “factor Xa” OR “factor-Xa”) AND (“vitamin K antagonist” OR “vitamin-K antagonist” OR “warfarin” OR “VKA” OR “OAC” OR “coumarin” OR “acenocoumarol”).

For comprehensiveness, we also examined the reference lists of eligible studies. The search was performed in September of 2021; therefore, articles published before this date were selected for the further selection process.

### 2.2. Inclusion Criteria

As few randomized controlled trials are available on the subject, we also included cohort studies in our study. A publication was included in our study if it met the following criteria: 1. the study contained a direct comparison of DOACs and VKA anticoagulants; 2. participants were adult patients (>18 years) suffering from PAD; 3. participants received anticoagulant, more frequently for NVAF or after a lower-extremity arterial revascularization procedure (open surgery or endovascular intervention) to improve patency of the bypass graft or due to poor runoff; 4. patients in the intervention group were receiving DOACs (rivaroxaban, apixaban, dabigatran, edoxaban); 5. patients in the control group were treated with VKA anticoagulant drugs (acenocoumarol, warfarin); 6. the study aimed to compare two types of oral anticoagulants; 7. the study contained at least one of the following outcomes: acute limb ischemia, major amputation, myocardial infarction, ischemic stroke, cardiovascular mortality, or all-cause mortality (efficacy indicators); and/or major bleeding (safety indicator).

### 2.3. Exclusion Criteria

We excluded ongoing trials without results, animal studies, conference abstracts, editorials, case reports, and studies with no original data.

### 2.4. Data Extraction

The publications collected from the databases were screened by two independent authors (E.P. and B.K.). After duplicate removal, publications were screened first by title and abstract, then by full text. Disagreements were resolved by consensus. From each article, we extracted for further analysis important data, such as basic information (author, year of publication, study design, and number of centers); study characteristics (population size, follow-up time); demographic characteristics of the patients involved, such as age, gender, relevant comorbidities, and medication; type and dose of DOACs and VKA; and outcomes of interest (major adverse limb events, incidence of revascularization procedures and amputation, incidence of myocardial infarction, ischemic stroke during follow-up time, all-cause, and cardiovascular mortality). Adjusted hazard ratios (HRs) or propensity-score-matched HRs with 95% confidence intervals (CIs) or calculated odds ratios (ORs) were extracted as measures of effect.

### 2.5. Quality Assessment

**Figure 1:**
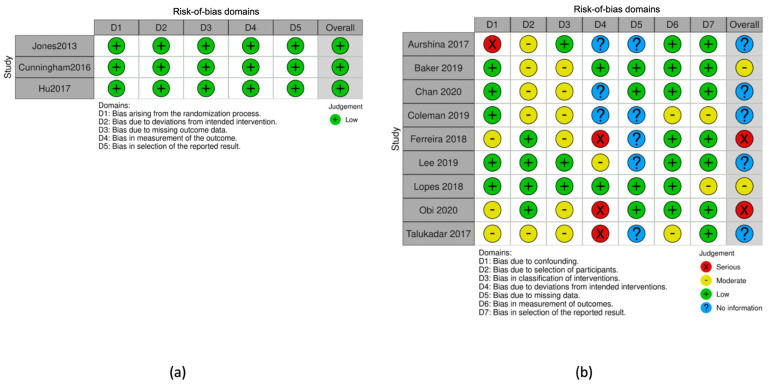
Risk-of-bias assessment and overall risk of each type of bias using (**a**) Cochrane Collaboration tool in RCTs and (**b**) ROBINS-I tool in observational prognostic studies.

The Cochrane Collaboration tool (Rob-2) was used to assess the risk of bias for the three post hoc analyses of RCTs and the ROBINS-I tool for the non-randomized studies of interventions [23,24]. The results of the risk-of-bias assessment are summarized in Figure 1.

### 2.6. Statistical Analysis

For the selected groups, either hazard ratios (HR) or raw patient numbers were extracted and analyzed. For dichotomous outcomes, the odds ratio (OR) with 95% confidence intervals (CI) was used to measure effect. Raw data from the selected studies were pooled using a random-effects model. We estimated the τ^2^ using restricted maximum likelihood approach, and the Q-profile method for calculating the confidence interval of τ^2^. Statistical heterogeneity across trials was assessed using the Cochrane Q-test and the I^2^ values. Subgroup analyses followed the descriptions of Harrer et al. [25]. Outlier and influence analyses were performed following the recommendations of Harrer et al. [25], and Viechtbauer and Cheung [26].

All statistical analyses were performed in R (R Core Team 2021, v4.1.2) using the *meta* (Schwarzer 2021, v5.1.1) and *dmetar* (Cuijpers, Furukawa, and Ebert 2021, v0.0.9000) packages [27,28,29].

## 3. Results

**Figure 2:**
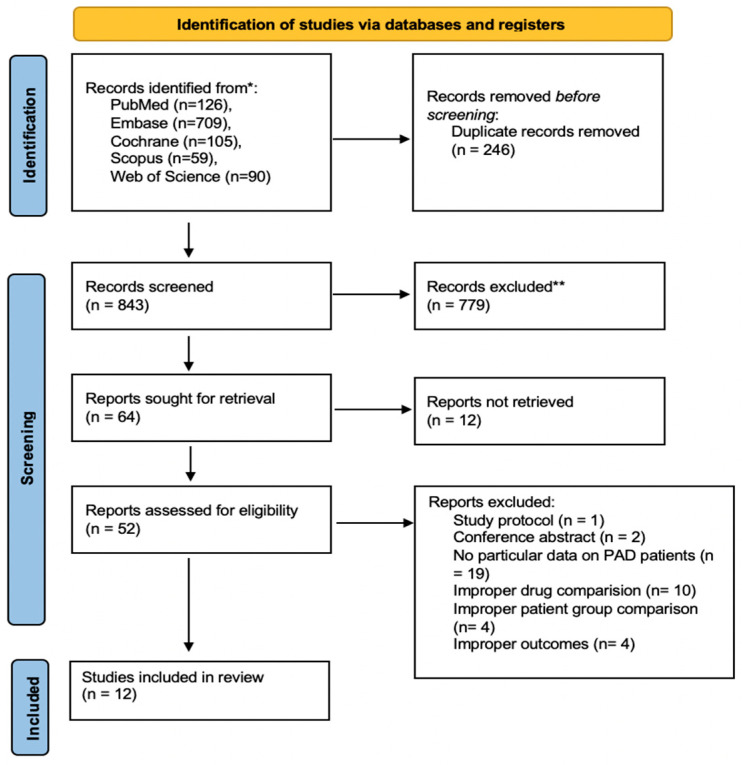
The PRISMA flowchart of the article selection process.

The literature search yielded a total of 1089 articles. During the selection procedures, 52 full-text articles were screened for eligibility, of which a total of 12 articles (three post hoc analyses from big randomized controlled trials [30,31,32], and nine observational cohorts [33,34,35,36,37,38,39,40,41]) were finally included (Figure 2).

The original articles were published between 2013 and 2020, and data were collected between 2006 and 2017. Important information on the included studies and the basic characteristics and demographic data of the included patients is shown in Table 1 and Table 2. 

**Figure 3:**
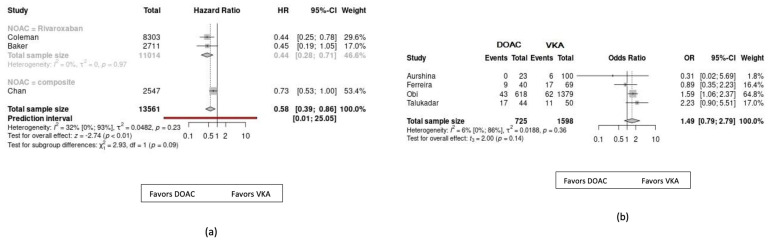

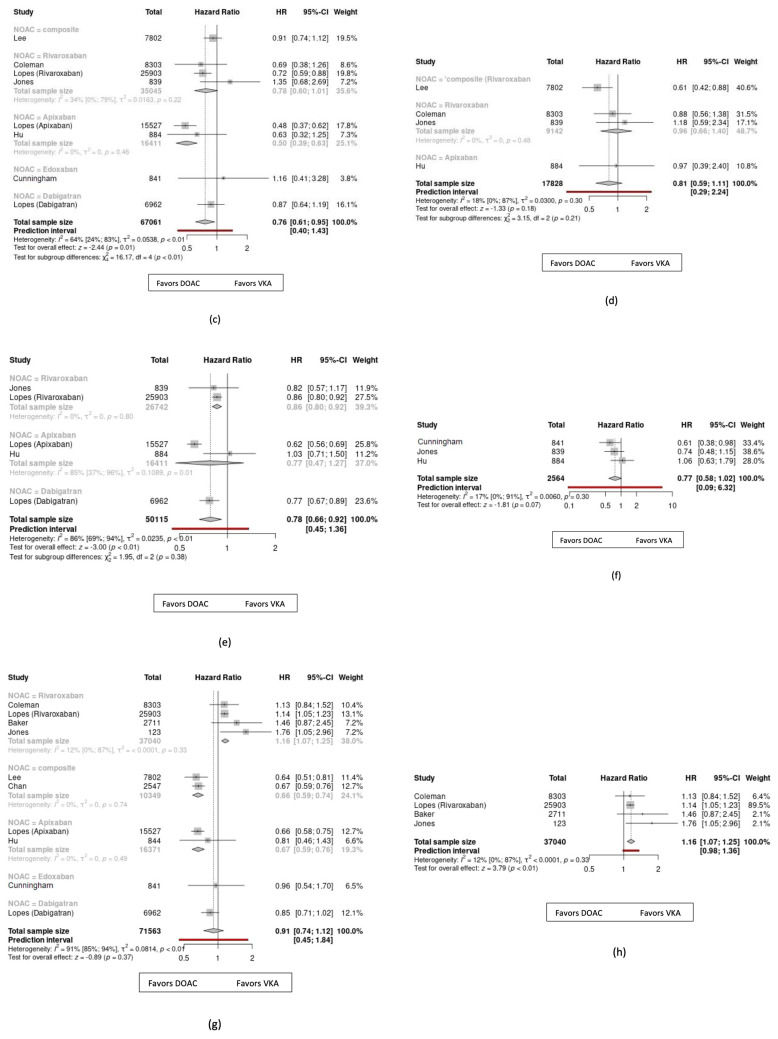

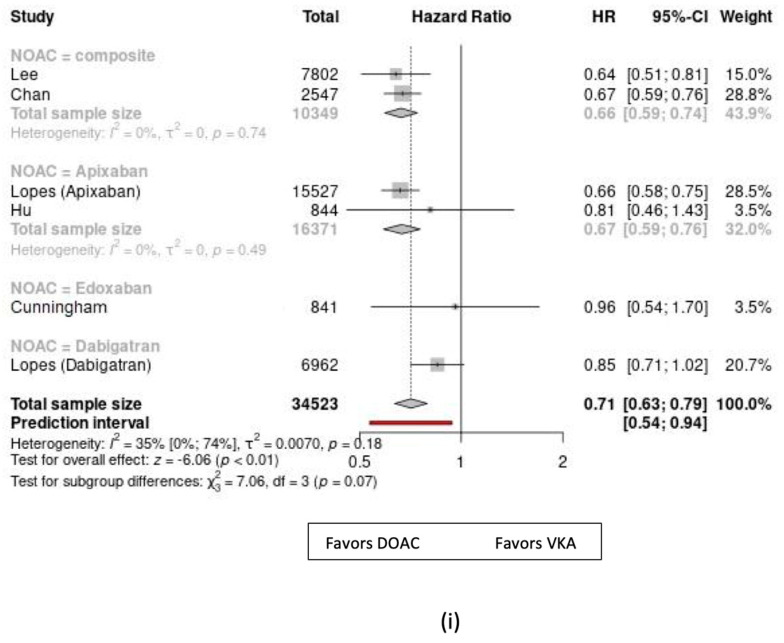
Results: (**a**) major adverse limb events (MALE); (**b**) need for revascularization; (**c**) stroke/systemic embolism; (**d**) myocardial infarction; (**e**) all-cause mortality; (**f**) cardiovascular mortality; (**g**) major bleeding (all DOACs); (**h**) major bleeding–rivaroxaban (15/20 mg daily); (**i**) major bleeding–apixaban, dabigatran, edoxaban at conventional doses, and rivaroxaban at reduced doses (2.5 or 5 mg daily).

All results are presented in Table 3 and Figure 3. To assess the strength of recommendations for each outcome, we used the online GRADEpro tool (https://www.gradepro.org), which is based on the GRADE (Grading, Development and Evaluation of Recommendations) method, providing a useful way of assessing the quality of evidence [42], (Table 3).

Our primary point of interest is the lower-limb events, which are reported as a composite outcome of major adverse limb events (MALE). It is defined as a summary of lower-limb revascularization and amputation events. We analyzed this outcome separately, according to whether the indication for anticoagulation was the concomitant NVAF or the aim to improve the patency after an open or endovascular lower-extremity arterial procedure. 

Three articles contained this outcome, which involved 13,561 PAD patients who received anticoagulation therapy because of their concomitant NVAF. All three articles used propensity-score-matched data. Our results showed that patients in the DOAC group were significantly less likely to experience a MALE during the study period than patients in the VKA group, with moderate heterogeneity (HR = 0.58, 95% CI, 0.39–0.86, *p* < 0.01, I^2^: 32%).

We found four observational studies on PAD patients without AF, where the need for reoperation was a separate outcome. In this case, the data of 2323 patients were available, and we found no significant difference between the two anticoagulant groups, without any evidence of heterogeneity (OR = 1.49, 95% CI, 0.79–2.79, *p* = 0.14, I^2^: 6%). Unfortunately, there were only a few data available about the need for amputation; therefore, no statistical analysis was applicable. 

We found data regarding the other efficacy outcomes on patients with PAD and AF. In this patient population, compared with VKAs, the use of DOACs was associated with a significantly reduced risk of stroke/systemic embolism (SE) (HR 0.76; 95% CI 0.61–0.95; *p* < 0.01; I^2^ = 64%) and all-cause mortality (HR 0.78; 95% CI 0.66–0.92; *p* < 0.01; I^2^ = 86%), with substantial heterogeneity. We did not find a statistically significant difference between the DOAC and VKA groups in terms of the incidence of myocardial infarction (HR = 0.81, 95% CI, 0.59–1.11, *p* = 0.21, I^2^: 18%) and cardiovascular mortality (HR = 0.77, 95% CI, 0.58–1.02, *p* = 0.07, I^2^: 17).

As for the safety of the anticoagulant medication, major bleeding was observed in most of the studies on PAD patients with AF. Comparing all the DOACs with the VKAs, we found similar risks (HR = 0.91, 95% CI, 0.74–1.12, *p* < 0.01, I^2^: 91%) for the occurrence of major bleeding episodes. For this outcome, we analyzed the results separately due to the considerable heterogeneity, and found that rivaroxaban at higher doses significantly increased the risk of bleeding (HR = 1.16, 95% CI, 1.07–1.25, *p* < 0.01, I^2^: 12%); on the other hand, we found a significantly lower risk of major bleeding in the composite group of the other three DOAC drugs at conventional dosages and rivaroxaban administered at a reduced dose (HR = 0.71, 95% CI, 0.63–0.79, *p* < 0.01, I^2^: 35%).

## 4. Discussion 

In patients with atrial fibrillation and PAD, optimal anticoagulation is of key importance, but the progression of atherosclerosis as the underlying disease also needs to be considered. Apart from this study, no other study has attempted to directly compare patients who take anticoagulants after revascularization. 

Our results show that, in patients with NVAF and concomitant PAD, the incidence of MALE was significantly lower in DOAC users compared with VKA users. In the three articles reporting this composite outcome, rivaroxaban was compared with warfarin at the same doses. Coleman et al. described [36] that rivaroxaban was associated with a significant reduction in the risk of major thrombotic vascular events (MTVEs), including cardiovascular events, as well as major adverse limb events. Baker et al. and Chan et al. mainly studied NVAF patients with diabetes [34,35]. In all the participants of their studies as well as in the subgroup of PAD patients, rivaroxaban was associated with a significantly lower risk of MALE, and this effect was due to a reduction in the risk of both major limb amputation and endovascular revascularization; however, the risk of surgical revascularization did not differ between the groups. In a nationwide retrospective cohort study from Taiwan [38], the authors analyzed the data of 7802 AF patients with concomitant PAD who were receiving anticoagulant medication. According to their results, all the DOACs (dabigatran, rivaroxaban, apixaban, and edoxaban) were also associated with a lower cumulative risk of lower-limb embolization or amputation and revascularization procedures compared with warfarin.

According to our findings, the use of DOACs in AF patients with PAD was associated with a significantly reduced risk of stroke/STE and all-cause mortality, but we did not find a statistically significant difference in terms of CV mortality and MI. Baker et al. reported results similar to ours [34], stating that AF patients who received a reduced dose of rivaroxaban compared with warfarin had significantly decreased the rates of MALE but not of major adverse cardiac events (MACE).

According to Lee et al., PAD patients with AF had similar rates of ischemic stroke with rivaroxaban, but a significantly lower annual incidence of acute myocardial infarction (MI) with warfarin [38]. In the study by Lopes et al. [39], all DOACs were associated with lower stroke/MI/all-cause mortality rates compared with warfarin. The studies by Hu and Jones reported data on MI, finding no significant difference in MI risk and CV mortality between the DOAC and warfarin groups. However, in the Cunningham study, the risk of CV mortality was higher with high-dose edoxaban than with warfarin [30,31,32].

In a meta-analysis examining the relationship between AF and PAD, Zhu et al. stated [43] that the occurrence of PAD in patients with AF could increase the risk and incidence of several adverse clinical events, including all-cause mortality, cardiovascular (CV) death and MACE; however, they did not find a statistically significant difference in the incidence of major bleeding, myocardial infarction (MI), and stroke among AF patients with and without PAD.

Another meta-analysis by Liao et al. found [44] similar thromboembolic and bleeding risks in AF patients with and without PAD, but patients with PAD had an increased risk of death compared with those without it. In their ROCKET AF trial, Pokorney et al. examined patients who were anticoagulated for NVAF [45], and collected factors associated with a higher risk of mortality. They found that PAD, heart failure, and diabetes were most strongly associated with a higher likelihood of CV death. 

In PAD patients without atrial fibrillation and no other indication for anticoagulation but to improve graft patency after lower-extremity surgery, Kretschmer et al. already made a comparison between the postoperative use of antiplatelet (aspirin) and VKA anticoagulants in 1992 [46]. Twenty years later, the Dutch BOA study and several other researches confirmed their original conclusion that VKA treatment is associated with improved graft-patency rates when a vein graft was used, while there is no difference with prosthetic grafts. However, patients receiving an artificial graft might profit more from platelet inhibitors [47]. Recent guidelines [2,3,4,5,6] also suggest that, after endovascular revascularization, a period of combination therapy of anticoagulants and antiplatelets should be considered bearing in mind the bleeding and thrombotic risks, but the period of this combination therapy should be as brief as possible.

Regarding the appropriate antiplatelet and/or anticoagulant therapy in PAD patients, two large randomized controlled trials carried out in recent years have emphasized the beneficial effects of aspirin and low-dose rivaroxaban in the prevention of cardiovascular consequences and lower-extremity events compared with the use of aspirin alone or a higher dose of rivaroxaban alone [48,49,50]. 

Smith et al. highlighted [51] that the overall use of anticoagulants increased to one-third of all below-the-knee bypasses secondary to the greater use of DOACs. This is due to the widespread adaptation after the publication of the COMPASS trial. 

Currently, in our study, we have only found four single-center retrospective studies with a small number of cases directly comparing the two anticoagulant groups (DOAC vs. VKA) in terms of their postoperative use. No larger clinical trial or review is available. We found no significant overall difference in the need for reoperation between patients who were prescribed DOAC or VKA postoperatively. Talukadar et al. found the safety and efficacy profile of rivaroxaban to be comparable to that of warfarin when used in patients after peripheral arterial procedures [41]. The results of Aurshina et al. suggest [33] that therapy with DOACs has an excellent graft primary patency rate at one-year follow-up. According to Ferreira et al. [37], rivaroxaban has equivalent efficacy to acenocoumarol after infrainguinal bypass revascularization, with similar rates of occlusion, major amputation, and mortality. In addition, Obi et al. found in patients undergoing lower-extremity surgical bypass that those receiving DOAC postoperatively had a shorter length of stay and were less likely to receive a transfusion in the following 30 days without compromising the graft patency and readmission rates for anticoagulation complications, thrombectomy, or thrombolysis, or affecting the amputation rate compared with those receiving a VKA [40].

On the basis of the results of these smaller-volume retrospective cohorts, DOACs and VKAs are likely to show equivalent or similar patency and amputation rates following revascularization [33,37,40,41].

Another important issue about anticoagulation is its safety. Although there is almost no difference in the efficacy in preventing thromboembolic complications, there are significant differences in the safety profile of the anticoagulants. In our study, we found that rivaroxaban at a daily dose of 15 or 20 mg significantly increased the risk of bleeding compared with VKA. On the other hand, we found a significantly lower risk of major bleeding episodes in users of the other three DOAC drugs, or even with rivaroxaban at lower doses (2.5 or 5 mg per day). Ingason et al. found [52] that the use of rivaroxaban was associated with higher overall rates of gastrointestinal bleeding (GIB) compared with apixaban and dabigatran in patients with AF. Wang et al. reported [53] that AF patients taking apixaban and dabigatran, but not rivaroxaban, experienced fewer bleeding events compared with warfarin. Radadiya et al. performed a network meta-analysis including 28 RCTs and 139,587 patients. In the study, DOACs at a standard dose, rivaroxaban at 20 mg daily, dabigatran at 150 mg twice daily, and edoxaban at 60 mg daily, but not apixaban at 5 mg twice daily, had a higher risk of major GIB compared with warfarin. The comparison of DOACs with each other did not show risk differences [54]. Numerous other authors state that rivaroxaban should be treated with caution, especially at higher doses, which is also supported by our results. Jones described that NVAF patients with PAD had a higher risk of experiencing a major bleeding episode than those without PAD [31]. We believe that the higher risk of bleeding is due to the concomitant antiplatelet drug usage independently of the anticoagulant dose in PAD patients. Chan et al. also suggest in their study [35] that they also found a lower risk of major bleeding for DOACs compared with warfarin, especially in patients who did not take concomitant antiplatelet medication besides the anticoagulation. Moreover, it is also important to highlight that patients at a higher risk of thromboembolic events are also at a higher risk of bleeding. In their systematic review and meta-analysis, Almas et al. assessed the safety and efficacy of DOACs with and without acetylsalicylic acid (ASA). The risk of major bleeding was significantly lower in the DOAC-alone group compared with the DOAC-plus-ASA group [55]. In the current literature, apixaban appears to have the best safety profile for bleeding among the four available DOACs, with similar efficacy to warfarin for stroke/SE [56,57,58,59].

We believe that all our findings provide useful information to help to select the optimal anticoagulant, although efficacy and bleeding risk should be carefully evaluated, especially in the presence of comorbidities such as peripheral artery disease.

## 5. Strengths and Limitations

We acknowledge the strengths and limitations of this meta-analysis.

The strength of the study is that it involves thousands of patients. In these cohorts, propensity-score-matched data are available. Our study provides useful findings on lower-limb outcomes and summarizes the latest results and recommendations.

The limitations of the study include the fact that residual confounding results are due to unmeasured factors, such as the lack of an international normalized ratio (INR) for patients treated with warfarin, the body weight, and the accurate renal function data which may have affected the validity of our findings. There could be a misclassification or miscoding of baseline comorbidities as well as potential bias due to different coding systems in different countries. The nomenclature is also not uniform: there is a significant overlap between the meanings of coronary artery disease (CAD), lower-extremity artery disease (LEAD), and PAD. Data reporting in a non-uniform format makes statistical analysis difficult. The use of different drug doses and differences in patient follow-up times lead to difficulties in the proper analysis of the data. Due to the limited data, we were not able to perform a subgroup analysis by age, comorbidities, or medication.

## 6. Conclusions

We have pointed out that, based on the present meta-analysis, the use of DOACs versus VKAs in PAD patients with NVAF is associated with significantly better outcomes in terms of major limb events, stroke, and mortality. Rivaroxaban at conventional doses increases the risk of major bleeding compared with other DOAC drugs. On the other hand, DOAC and VKA seem to produce equivalent or similar patency rates following infrainguinal revascularization procedures, but there is an absence of strong evidence.

Using DOACs in patients undergoing lower-extremity arterial procedures may play a more significant role in the future, but further investigations are needed for definitive results and safe decision-making.

## Figures and Tables

**Table 1:** Characteristics of the included studies: CV—cardiovascular, DOAC—direct-acting oral anticoagulant, INR—international normalized ratio, MALE—major adverse limb event, MI—myocardial infarction, NI—no information, NVAF—non-valvular atrial fibrillation, PAD—peripheral artery disease, RCT—randomized controlled trial, SE—systemic embolism, VKA—vitamin-K antagonist; * In this study, there were 66%, 89%, 68%, and 95% patients taking low-dose apixaban (2.5 mg twice daily), dabigatran (110 mg twice daily), edoxaban (30 mg daily), and rivaroxaban (15/10 mg daily); ** In this study, there were 89% patients taking low-dose NOAC (dabigatran 110 mg twice daily, rivaroxaban 10–15 mg daily, apixaban 2.5 mg twice daily, or edoxaban 30 mg daily).

First Author	Year of Publication	Study Design	Study Population/ Indication for Anticoagualtion	DOAC Type	DOAC Dose	VKA Type (Dosed According to INR Is in Therapeutic Range)	Outcomes
Aurshina [33]	2020	Retrospective cohort	PAD patients after revascularization	Rivaroxaban, Apixaban, Dabigatran	NI NI NI	Warfarin	Revascularization
Baker [34]	2018	Retrospective cohort (propensity score matched)	AF patients with PAD	Rivaroxaban	15/20 mg daily	Warfarin	Major bleeding, MALE
Chan * [35]	2020	Retrospective cohort (propensity score matched)	AF patients with PAD	Dabigatran, Rivaroxaban, Apixaban, Edoxaban	110/150 mg twice daily 10/15/20 mg daily 2,5/5 mg twice daily 30/60 mg daily	Warfarin	Major bleeding, MALE
Coleman [36]	2019	Retrospective cohort (propensity score matched)	AF patients with PAD	Rivaroxaban	15/20 mg daily	Warfarin	MI, Stroke/SE, Major bleeding, MALE
Cunningham [32]	2016	RCT (post hoc analysis)	AF patients with PAD	Edoxaban	30/60 mg	Warfarin	Stroke/SE, Major bleeding, CV-Mortality
Ferreira [37]	2017	Retrospective cohort	PAD patients after revascularization	Rivaroxaban	20 mg daily	Warfarin	Mortality, Amputation, Revascularization
Hu [30]	2013	RCT (post hoc analysis)	AF patients with PAD	Apixaban	5 mg twice daily	Warfarin	MI, Stroke/SE, Major bleeding, Mortality, CV-Mortality
Jones [31]	2018	RCT (post hoc analysis)	AF patients with PAD	Rivaroxaban	15/20 mg daily	Warfarin	MI, Stroke/SE, Major bleeding, Mortality, CV-Mortality
Lee ** [38]	2017	Retrospective cohort (propensity score matched)	AF patients with PAD	Dabigatran, Rivaroxaban, Apixaban, Edoxaban	110/150 mg twice daily 10/15/20 mg daily 2,5/5 mg twice daily 30/60 mg daily	Warfarin	MI, Stroke/SE, Major bleeding, Amputation, Revascularization
Lopes [39]	2018	Retrospective cohort (propensity score matched)	AF patients with PAD	Rivaroxaban, Dabigatran, Apixaban	10/15/20 mg daily 75/150 mg twice daily 2.5/5 mg twice daily	Warfarin	Stroke/SE, Major bleeding, Mortality
Obi [40]	2018	Retrospective cohort	PAD patients after revascularization	Not specified	NI	Not specified	Amputation, Revascularization Mortality
Talukadar [41]	2019	Retrospective cohort	PAD patients after revascularization	Rivaroxaban	NI	Warfarin	Major bleeding, Revascularization

**Table 2:** Basic characteristics and patient demographics; parameters expressed as mean with standard deviation (*), or median with interquartile range (**); DOAC—direct-acting oral anticoagulant, NI—no information, VKA—vitamin-K antagonist.

First Author	Year of Publication	Age (All) (Years)	DOAC Age (Years)	VKA Age (Years)	MALE (*n*)	Hypertonia (*n*)	Diabetes (*n*)	Ischaemic Heart Disease (*n*)	Cerebrovascular Disease (*n*)	Chronic Renal Disease (*n*)	Antiplatelet Use (*n*)	Statin Use (*n*)	DOAC Patient Number (*n*)	VKA Patient Number (*n*)	Follow-Up Time (Months)
Aurshina [33]	2017	NI	69 ± 11 *	72 ± 12 *	61	101	82	NI	NI	NI	NI	NI	23	100	23 ± 16 *
Baker [34]	2019	NI	NI	NI	NI	NI	NI	NI	NI	NI	NI	NI	NI	NI	16.8 (7.2–32.4) **
Chan [35]	2020	NI	NI	NI	NI	NI	NI	NI	NI	NI	NI	NI	NI	NI	NI
Coleman [36]	2019	74 (65–81) **	NI	NI	5307	7144	3819	3902	913	2740	2325	5314	3257	5046	16.8 (7.2–32.4) **
Cunningham [32]	2016	NI	NI	NI	NI	NI	NI	NI	NI	NI	NI	NI	NI	NI	NI
Ferreira [37]	2018	64.8 ±NI *	64.4 ± NI *	65 ± NI *	86	81	65	36	19	29	84	NI	40	69	12
Hu [30]	2017	73 (66.5–79) **	NI	NI	593	813	325	523	295	NI	378	NI	442	442	12
Jones [31]	2013	74 (67–79) **	NI	NI	606	768	400	289	480	NI	346	NI	401	438	24.33 (19.2–29.9) **
Lee [38]	2019	NI	77.4 ± 9.7 *	77.3 ± 9.9 *	4410	7115	4090	1379	1665	3226	NI	964	5768	2034	NI
Lopes [39]	2018	NI	78.9 ± 7.5 *	79 ± 7.5 *	16,930	29,455	13,160	18,634	8327	8475	8621	22,396	15,527	15,527	5–6
		NI	77.8 ± 7.1 *	78.2 ± 7.3*	7708	13,083	6332	8393	3450	3305	3370	9503	6962	6962	5–6
		NI	78.3 ± 7.4 *	78.5 ± 7.4 *	28,440	48,845	22,300	31,101	13,044	13,054	12,811	35,699	25,903	25,903	5–6
Obi [40]	2020	NI	66.5 (57.5–74.4) **	65.4 (58–73) **	1329	1773	811	1044	564	79	1634	NI	1379	618	12
Talukadar [41]	2017	NI	60.5 ± 15 *	63.8 ± 14 *	59	NI	28	NI	5	NI	78	NI	44	50	NI

**Table 3:** Summary of findings table; CI—confidence interval, DOAC—direct-acting oral anticoagulant, HR—hazard ratio, OR—odds ratio, PAD—peripheral artery disease, RCT—randomized controlled trial.

Outcome	Study Numbers and Type	Number of Patients Involved	Relative Effect (95% CI)	Quality (GRADEpro)	Comments
MALE	3 Cohorts	13,561	**HR = 0.58**; (0.39–0.86); *p* < 0.01	⨁⨁⨁⨁ High	Composite outcome of reoperation and amputation
Need for revascularization	4 Cohorts	2323	**OR = 1.49**; (0.79–2.79); *p* = 0.14	⨁⨁ Low	The outcome was observed in PAD patients who were prescribed anticoagulants after arterial revascularization procedure
Myocardial infarction	3 RCTs	17,828	**HR = 0.81**; (0.59–1.11); *p* = 0.21	⨁⨁⨁⨁ High	
Stroke/systemic embolism	3 RCTs 3 Cohorts	67,061	**HR = 0.76**; (0.61–0.95); *p* < 0.01	⨁⨁⨁ Moderate	
All-cause mortality	2 RCTs 1 Cohorts	50,115	**HR = 0.78**; (0.66–0.92); *p* < 0.01	⨁⨁⨁ Moderate	
Cardiovascular mortality	3 RCTs	2564	**HR = 0.77**; (0.58–1.02); *p* = 0.07	⨁⨁⨁⨁ High	
Major bleeding	3 RCTs 5 Cohorts	71,563	**HR = 0.91**; (0.74–1.12); *p* < 0.01	⨁⨁⨁⨁ High	The outcome was observed with high-dose Rivaroxaban (10/20 mg daily) in 34,523 patients (HR = 1.16, 1.07–1.25, *p* < 0.01); and with other NOAC and low-dose Rivaroxaban (2.5 or 5 mg daily) in 37,040 patients - (HR = 0.71, 0.63–0.79, *p* < 0.01)

## Data Availability

Data are contained within the article.

## Sample Availability

Samples of the compounds are available from the authors.
